# Analysis of Phenolic Compounds and Antioxidant Abilities of Extracts from Germinating *Vitis californica* Seeds Submitted to Cold Stress Conditions and Recovery after the Stress

**DOI:** 10.3390/ijms150916211

**Published:** 2014-09-12

**Authors:** Stanisław Weidner, Sebastian Chrzanowski, Magdalena Karamać, Angelika Król, Anna Badowiec, Agnieszka Mostek, Ryszard Amarowicz

**Affiliations:** 1 Department of Biochemistry, University of Warmia and Mazury, Oczapowskiego Street 1A, 10-957 Olsztyn, Poland; E-Mails: weidner@uwm.edu.pl (S.W.); chrzanowskiseb@gmail.com (S.C.); angelikakrol@op.pl (A.K.); anna@badowiec.eu (A.B.); aga.mostek@wp.pl (A.M.); 2 Institute of Animal Reproduction and Food Research of the Polish Academy of Sciences, Tuwima Street 10, 10-748 Olsztyn, Poland; E-Mail: m.karamac@pan.olsztyn.pl

**Keywords:** grape seeds, germination, chill stress, phenolic compounds, antioxidant activity

## Abstract

The material for this study consisted of stratified seeds of *Vitis californica* submitted to germination under optimum conditions (+25 °C) or under chill stress (+10 °C), also followed by recovery. It has been determined that the germinating seeds contain considerable amounts of tannins, catechins as well as phenolic acids such as gallic, *p*-coumaric, caffeic and ferulic acids. Gallic acid appeared in the highest amount in the germinating seeds (from 42.40–204.00 µg/g of fresh weight (FW)), followed by caffeic acid (from 6.62–20.13 µg/g FW), *p*-coumaric acid (from 2.59–5.41 µg/g FW), and ferulic acid (from 0.56–0.92 µg/g FW). The phenolic acids occurred mostly in the ester form. Under chill stress, the germinating seeds were determined to contain an elevated total amount of phenolics, as well as raised levels of condensed tannins, catechins, gallic acid, and gafeic acid. The levels of *p*-coumoric and ferulic acids were found to have decreased. In extracts isolated from a sample exposed to low temperature, increased antioxidant activity and reduction potential were also demonstrated. Tissue of the germinating seeds which underwent post-stress recovery was found to have less total phenolics.

## 1. Introduction

Plants growing under natural conditions are constantly exposed to a variety of stress factors. Their ability to thrive in a given environment, develop as an individual, produce offspring and generate yield depends on how adaptable they are to living in this environment. Plants have managed to develop complex mechanisms which help them to maintain homeostasis and to resist—to different degrees—stress factors which appear in nature. 

Among the major abiotic stress-inducing factors are: extreme temperatures, water deficit or excessive salinity. With respect to chill stress, it is assumed that it can be caused by temperatures below +12 °C. Plants sensitive to low temperatures are the ones which can experience irreversible changes at temperatures just below +12 °C, whereas the ones tolerant to chill (but vulnerable to freezing temperatures) die when the temperature falls below zero [1].

On the one hand, oxygen enables cells to acquire much more energy by respiration than supplied during fermentation processes. On the other hand, it can damage the cellular constituents by forming reactive oxygen species (ROS) [2]. ROS are capable of damaging DNA, proteins, lipids and chlorophyll [3]. Among the most frequently reported ROS are superoxide radical, hydrogen peroxide and hydroxyl radical. Most of the reactive oxygen forms in animal cells are produced in mitochondria in the respiratory chain. In plant cells, an important additional source of superoxide anion radicals is chloroplasts [2]. When the level of ROS in cells increases excessively, oxidative stress appears, which is a common and shared feature of all types of stress [4].

Repair antioxidative systems have developed in aerobic organisms during evolution, as an adaptation to the threat posed by ROS. These are mainly enzymatic systems, including superoxide dismutase (DOS), catalase, glutathione peroxidase, glutathione reductase, glucose-6-phosphate dehydrogenase as well as non-enzymatic mechanisms [5]. The non-enzymatic mechanisms encompass low-molecule organic substances which function as free radical traps, e.g., β-carotene, α-tocopherol, ascorbate, glutamate as well as numerous phenolic compounds [2,6]. The antioxidant properties of phenolic compounds depend on such factors as the structure of these compounds, their concentration, oxidation-reduction potential and hydrophilicity [6,7]. It is worth mentioning that individual phenolics show their characteristic antioxidant capacities, while a mixture containing two or three combined phenolics demonstrates the antioxidant capacity which is the sum of the individual capacities of the phenolics [7]. Therefore, there is no synergistic effect between the examined phenolics, but just an additive effect of the individual antioxidant capacities observed in the cited research. 

The biological material for the present study consisted of seeds of *Vitis californica* (California wild grape). Noteworthy is the fact that the wild grape is strong and robust so viticulturists often use it as root-stock for their wine grapes. So, grapevine is a widely bred species with an undeniable value for development of agriculture. However, its sensitivity to low temperatures in early growing may be troublesome in the many. Unfortunately, in the literature too little attention is paid to the role of phenolic compounds in a situation of chill stress affecting young plants, which are most sensitive to its influence. The major aim of the study has been to analyse quantities and composition of phenolic compounds as well as properties of antioxidants in seeds of *Vitis californica* germinating under chill stress. Another question examined was the effect of post-stress recovery on changes in the secondary metabolism of germinating seeds of *Vitis californica.*

## 2. Results and Discussion

### 2.1. Content of Phenolic Compounds

**Figure 1:**
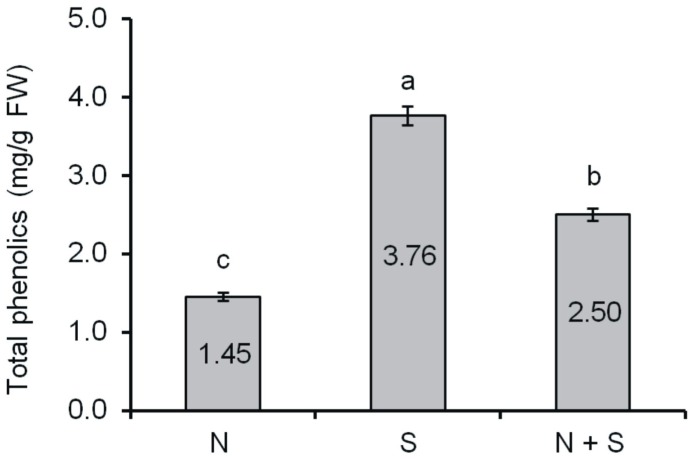
Content of total phenolics in grape seeds (mg/g of fresh weight). Means with the same letter (a,b,c) are not significantly different (*p* < 0.05).

The total content of phenolic compounds in seeds of *V. californica* germinating under different conditions is shown in Figure 1. The lowest total content of phenolics was determined in seeds germinating under optimum conditions (sample NS), where it reached 1.45 mg/g FW. Under chill stress (sample S), a distinct increase in the content of these compounds in germinating seeds was observable (3.76 mg/g FW). In turn, in seeds submitted to post-stress recovery (sample S + R), the content of total phenolics in germinating seeds declined to 2.50 mg/g FW.

The content of tannins in germinating *V. californica* seeds was analyzed with the vanillin assay and the bovine serum albumin (BSA) precipitation method. In all the extracts from germinating *V. californica* seeds examined with the vanillin assay, presence of tannins was detected (Table 1). The highest absorbance among all the tested seeds was detected in the ones germinating under chill stress (sample S), where it equalled 0.282 and was nearly twice as high as in extracts from seeds germinating under optimum conditions (sample NS), in which it was 0.147 A_500_/mg. The lowest value of absorbance was recorded for extracts from seeds germinating under post-stress recovery conditions (sample S + R), where it was 0.134 A_500_/mg of extract. Compared to seed germinating under chill stress (sample S), its value fell nearly two-fold. In extracts from seeds germinating under optimum conditions, under chill stress and submitted to recovery after stress, the content of tannins was also determined with the method of precipitating tannins from bovine serum albumin (BSA). In all the analysed extracts from germinating seeds, it was demonstrated that tannins were capable of binding with BSA, which resulted in their precipitation. The results obtained via the precipitation method proved to be similar to the results achieved by the vanillin assay. Similarly to the method discussed previously, the highest absorbance among all the samples was found in extracts from seeds germinating under chill stress (sample S). Its value was 0.384 A_510_/mg of extract, being more than double the absorbance attained for extracts from sample NS (0.153 A_510_/mg of extract), in which seeds were germinated under optimum conditions. In turn, much lower absorbance was detected in extracts from the sample derived from seeds germinating under chill stress followed by recovery (sample S + R), where it fell to 0.212 A_510_/mg of extract. Compared to the seeds germinating under chill stress (sample S), the absorbance decreased by nearly two-fold. 

**Table 1:** Content of condensed tannins in the extracts.

Group	Absorbance at 500 nm/mg Extract	Absorbance at 510 nm/mg Extract
NS	0.147 ± 0.005b	0.153 ± 0.014c
S	0.282 ± 0.008a	0.384 ± 0.010a
S + R	0.134 ± 0.003c	0.212 ± 0.013b

Means with the same letter (a,b,c) are not significantly different (*p* < 0.05).

The results of our analyses concerning the content of catechins in seeds of *V. californica* germinating under chill stress and submitted to post-stress recovery are shown in Table 2. Seeds germinating under chill stress were characterised by a higher content of catechins (sample S, 63.0 µg/g FW) than the ones germinating under optimum conditions (sample NS, 52.9 µg/g FW). When the chill stress was followed by a recovery period, the content of these compounds was considerably depressed (sample S + R, 40.8 µg/g FW). In all the samples, the share of epicatechin was much higher than that of catechin. The respective quantities of epicatechin and catechin (in µg/g FW) in the three types of samples was as follows: 32.0 and 20.9 in sample NS, 39.0 and 23.6 in sample S and 25.3 and 15.5 in sample S + R. 

**Table 2:** Content of catechins in seeds (μg/g FW).

Group	Catechin	Epicatecin	Catechin + Epicatechin
NS	20.9 ± 0.5b	32.0 ± 0.8b	52.9 ± 1.3b
S	23.6 ± 0.6a	39.4 ± 1.0a	63.0 ± 1.6a
S + R	15.5 ± 0.5c	25.3 ± 0.6c	40.8 ± 1.1c

Means with the same letter (a,b,c) are not significantly different (*p* < 0.05).

Further assays focused on fluctuations in the content of phenolic acids in grape seeds germinating under different conditions. The first compound to be analysed was gallic acid. In all the extracts from grape seeds, presence of gallic acid, both free and ester- or glycoside-bound one, was detected (Table 3). In seeds germinating under chill stress (sample S), the total content of gallic acid was demonstrably higher than in extracts from seeds germinating under optimum conditions (sample NS). The total content of gallic acid was 204.00 in sample S and 142.4 µg/g FW in sample NS. In contrast, the content of this acid was observed to have declined to 196.11 µg/g FW in seeds which underwent post-stress recovery (sample S + R). Gallic acid appeared mainly as an ester-bound compound. Tendencies in modifications of the content of this form of gallic acid in seeds germinating under different conditions resembled the changes observed in the total content of gallic acid in seeds. The content of gallic acid liberated from ester bonds in the analysed seed extract samples was the following: 31.56 in sample NS, 173.81 in sample S and 167.62 µg/g FW in sample S + R. In respect to the content of free gallic acid and its glycoside-bound form, it can be concluded that their quantities were small and ranged from 5.22–16.06 µg/g FW. 

**Table 3:** Content of phenolic acid in seeds (μg/g FW).

Species	Form of Phenolic Acids	Gallic Acid	Caffeic Acid	*p*-Coumaric Acid	Ferulic Acid
NS	Free	5.62 ± 0.01c	1.11 ± 0.01a	0.59 ± 0.01a	0.17 ± 0.01
	Esterified	31.56 ± 0.01c	5.51 ± 0.01c	3.67 ± 0.01b	0.75 ± 0.01a
	Glucosided	5.22 ± 0.03c	-	-	-
	Total	142.40 ± 0.04b	6.62 ± 0.02c	4.26 ± 0.02b	0.92 ± 0.02a
S	Free	15.22 ± 0.38a	0.36 ± 0.01b	0.08 ± 0.01b	trace
	Esterified	173.81 ± 4.34a	6.69 ± 0.18b	2.51 ± 0.01c	0.56 ± 0.01c
	Glucosided	14.97 ± 0.037b	-	-	-
	Total	204.00 ± 5.10a	7.06 ± 0.19b	2.59 ± 0.01c	0.56 ± 0.01c
S + R	Free	12.43 ± 0.31b	0.13 ± 0.01c	0.03 ± 0.01c	trace
	Esterified	167.62 ± 4.19b	17.17 ± 0.50a	5.38 ± 0.01a	0.62 ±0.01b
	Glucosided	16.06 ± 0.40a	-	-	-
	Total	196.11 ± 4.90a	20.13 ± 0.43a	5.41 ± 0.01a	0.62 ±0.01b

Means for the same form of phenolic acid with the same letter bovine (a,b,c) are not significantly different (*p* < 0.05).

In seeds germinating under chill stress, an increase in the content of caffeic acid from 6.62 (sample NS) to 7.06 µg/g FW (sample S) occurred. However, it was observed to increase up to 20.13 µg/g FW following post-stress recovery (sample S + R). Similarly to gallic and *p*-coumaric acids, the dominant form of caffeic acid in all the seed extracts was its ester-bound form. Tendencies in changes in the content of ester-bound caffeic acid were similar to the ones recorded for the total caffeic acid content. An increase in the ester-bound form of caffeic acid from 5.51 in sample NS to 7.06 µg/g FW in sample S was observed, with a subsequent rise up to 17.17 µg/g FW in the sample submitted to post-stress recovery (sample S + R). The free form of caffeic acid appeared (in germinating seeds) in very small amounts and gradually decreased in the subsequent samples. In sample NS, the content of this form of caffeic acid was the largest, *i.e*., 1.11; in sample S it reached 0.36 and in sample S + R it equalled 0.13 µg/g FW.

Comparing the content of *p*-coumaric in seeds germinating under chill stress (sample S) with its content in seeds germinating under optimum conditions (sample NS), it can be concluded that chill stress depressed the content of this acid in germinating seeds. The total content of *p*-coumaric acid in germinating seeds fell from 4.26 (optimum conditions) to 2.51 µg/g FW (chill stress). The post-stress recovery (sample S + R) induced an increase in the concentration of *p*-coumaric acid in germinating seeds up to 5.41 µg/g FW. The dominant fraction in all the samples, analogously to gallic acid, was the ester-bound form. In seeds germinating under stress conditions (sample S), a decrease in the content of *p*-coumaric acid liberated from ester bonds was noticed compared to the amount found in seeds germinating under optimum conditions, namely from 3.67 (sample NS) to 2.51 µg/g FW (sample S). During the recovery period after chill stress, an increase in the content of ester-bound *p*-coumaric acid appeared *versus* the seeds germinating under chill stress (sample S). After two days of incubation, the concentration of this form of *p*-coumaric acid grew to 5.41 µg/g FW (sample S + R). The free form of *p*-coumaric acid occurred in much lower concentrations. In response to chill stress, it fell from 0.59 (sample NS) to 0.08 µg/g FW (sample S). During the post-stress recovery period (sample S + R), the content of free *p*-coumaric acid continued to decrease down to 0.03 µg/g FW. No presence of glycoside-bound *p*-coumaric acid was detected in the analysed germinating seeds. 

The content of ferulic acid in the analysed seeds germinating under different conditions was the lowest of all the tested acids. The modifications of the content of ferulic acid in seeds germinating under optimum conditions, chill stress and chill stress followed by recovery were similar to the ones determined for *p*-coumaric and caffeic acids, discussed above. The total content of ferulic acid was 0.91 in sample NS, 0.57 in sample S and 0.62 µg/g FW in sample S + R. As in the case of gallic, *p*-coumaric and caffeic acids, the dominant fraction in all the seed extracts was the ester-bound form of ferulic acid. Changes in the ester-bound form of ferulic acid were the following: 0.75 in NS sample, 0.58 in S sample and 0.62 µg/g FW in S + R sample. Small amounts of free ferulic acid were determined only in sample NS (0.17 µg/g FW). No presence of glycoside-bound ferulic acid in germinating seeds of *V. californica* were found. 

Plants produce considerable amounts of secondary metabolites, among which phenolic compounds constitute an important group of substances. Phenolic compounds can be divided into several groups, distinguished by the number of constitutive carbon atoms in conjunction with the structure of the basic phenolic skeleton [6]. These compounds are claimed to play a variety of functions in plants. In the present research, it has been demonstrated that the total content of phenolic compounds in grape seeds germinating under chill stress can double. This may indicate that phenolics are engaged in the process of acclimation of plants to low temperatures. In seeds subjected to 48 h recovery after chill stress, a certain decrease in the amounts of phenolics in plant tissues was observed. However, their level was much higher than in the control sample (prior to stress). It is noteworthy that in lettuce a significant increase in the total phenolic content was noted immediately after heat shock (10 min) and chill stress (1 h) treatments [8]. This suggests that environmental shocks can readily increase the antioxidant activity in plants.

Among the phenolic compounds analysed in the germinating seeds of *Vitis californica*, the dominant ones were tannins and monomeric flavon-3-ols, that is catechins: (+) catechin and (−) epicatechin, which by polymerization are incorporated into proanthocyanidins, also known as condensed tannins. Similar results concerning grape seeds have been obtained by other authors [9,10,11,12,13,14,15,16,17,18]. The present experiment has demonstrated that the quantity of tannins was distinctly higher in seeds germinating under chill stress. It has been proven that these compounds are strong antioxidants [15,16] and can successfully scavenge free radicals [6,19,20]. An increase in the content of tannins in seeds of another grape species (*Vitis riparia*) germinating under chill stress was detected in some earlier study [18]. The same research showed that the content of tannins declines during seed stratification and in the first week of germinating seeds under optimum conditions. In the experiment described in this paper, the seeds germinating under chill stress have been observed to contain distinctly elevated levels of catechin and epicatechin. It is known that these compounds are the components of condensed tannins. Similar results were obtained in previous studies on grape seeds germinating under osmotic tress, where the said stress led to a considerable increase in the content of catechins (catechin + epicatechin) [21]. In the present article as well as in the paper cited above, epicatechin was demonstrated to dominate over catechin. An increase in the content of catechins and condensed tannins in grape seeds germinating under either osmotic or chill stress may confirm their important role in adapting plants to unfavourable environmental conditions. 

The final group of compounds identified in this paper were such phenolic acids as gallic, *p*-coumaric, caffeic and ferulic acids. For all the four acids, the prevailing form was the ester-bound one. With respect to *p*-coumaric, caffeic and ferulic acids, they were not detected to appear in analysed seed extracts in the glycoside-bound form. Estrification and glycolisation of phenolic aids, in addition to other factors, protect cells from adverse effects of the accumulation of these compounds [22]. Estrification of phenolic acids may also enable their transport to vacuoles [23]. In the present study, gallic acid appeared in the highest quantities in germinating grape seeds. Under chill stress, the content of all the three forms of this acid (free, ester- and glycoside-bound ones) was observed to increase. Solecka *et al*. [24] demonstrated that in winter oilseed rape leaves subjected to cold and then to freezing treatments, the levels of soluble *p*-coumaric, sinapic and ferulic acids increased about 3-, 4- and 5-fold, respectively. The same researchers also proved that high rates of accumulation of ferulic and sinapic acids under low temperatures were associated with their almost complete estrification. In another experiment performed on soybean roots, it has been revealed that exposure to low temperature induced an increase in the relative level of ester-bound-soluble phenolic acids and the highest increase was observed for ferulic acid [25]. Chalker-Scott and Fuchigami [26] provided evidence that an increase in phenolic production and their incorporation to cell walls either as suberin and lignin occurred under chill stress. However, it should be emphasized that the effect of phenolic acids on growth is a complex process and encompasses the metabolism of hormones, protein synthesis, membrane permeability as well as the impact on respiration and oxidative phosphorylation [27].

### 2.2. Antioxidant Activity

**Figure 2:**
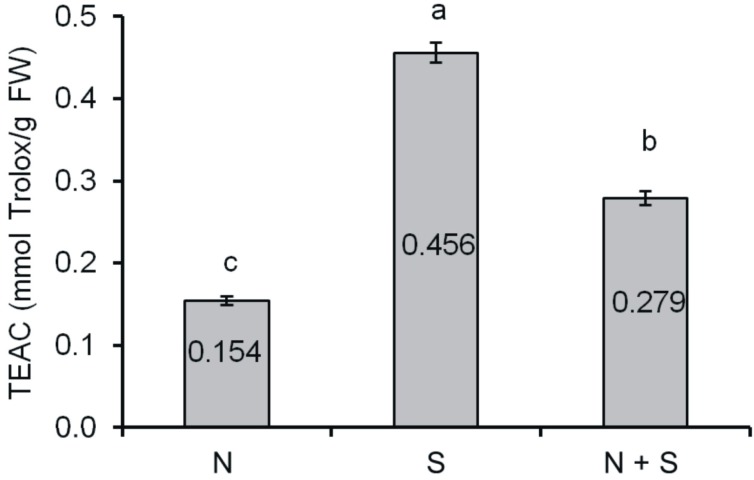
Trolox Equivalent Antioxidant Capacity (TEAC) of grape seeds (mmol Trolox/g FW). Means with the same letter (a,b,c) are not significantly different (*p* < 0.05).

The results which define the antioxidant capacity of grape seeds are presented in Trolox equivalents (Figure 2). The seeds germinating under chill stress (sample S) were characterised by stronger antioxidant capacity (0.456 mmol Trolox/g FW) than seeds germinating under optimum conditions (sample NS 0.154 mmol Trolox/g FW) or during recovery after stress (sample S + R 0.279 mmol Trolox/g FW). 

**Figure 3:**
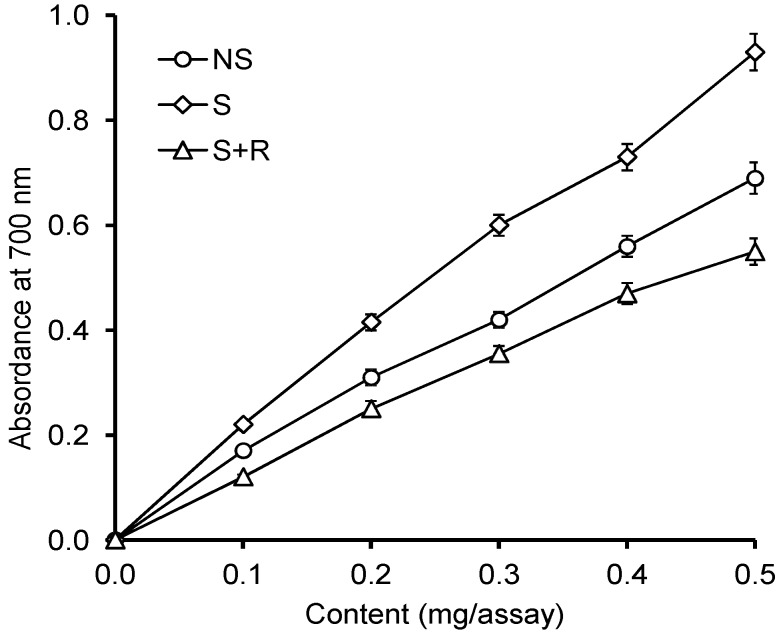
Reduction power of the extracts of grape seeds.

Under the influence of the reduction potential of the extracts, Fe^3+^ ions were reduced to Fe^2+^ ions, and the colour of the reaction mixture changed from yellow to various tints of blue and green. The content of the reduced Fe^2+^ ions was measured by analysing the amount of Prussian blue produced in the solution, using absorbance measurements at a 700 nm wavelength. The results are shown in Figure 3.

All the samples revealed reducing properties. The reduction potential of extracts from seeds germinating under chill stress (sample S) was much stronger than the reduction potential of extracts from seeds germinating under optimum conditions (sample NS) or submitted to recovery after stress (sample S + R). In sample S of the concentration of 0.5 mg of extract/sample, the value of absorbance at 700 nm was 0.931. The values of absorbance for samples NS and S + R were much lower and equalled 0.692 and 0.553, respectively. 

**Figure 4:**
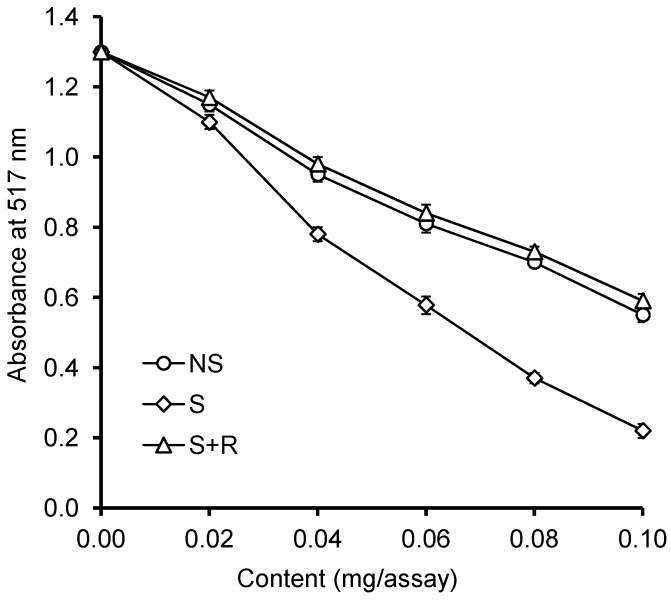
Antiradical activity of the grape seed extracts against 2,2'-diphenyl-1-picrylhydrazyl (DPPH) free radical.

The ability to scavenge a 2,2'-diphenyl-1-picrylhydrazyl (DPPH) free radical by extracts from germinating grape seeds is illustrated in Figure 4. This analytical method relies on using a DPPH• free radical (2.2-diphenyl-1-picrylhydrazyl), whose colour is purple of the maximum absorbance at 517 nm. When extracts from *V. californica* seeds are added to this solution, the radical is reduced, which leads to its being scavenged. As a result, the solution is gradually paler in colour and the value of absorbance decreases in analysed samples. Samples of the highest antioxidant capacity are characterised by the lowest absorbance values. In the present experiment, it has been observed that all the tested extracts from *V. californica* seeds were capable of scavenging the DPPH• free radical, but extracts from seeds which germinated under chill stress (sample S) were more powerful scavengers than extracts from seeds germinating under optimum conditions (sample NS) or the ones submitted to post-stress recovery (sample S + R). In sample S of the extract concentration equal 0.1 mg/sample, the value of absorbance at 517 nm was 0.221. The analogous values for samples NS and S + R were similar and equalled 0.554 and 0.591, respectively. 

Our further investigations presented in this paper involved determination of the antioxidant and reduction potential of extracts from germinating seeds of *Vitis californica*. It has been demonstrated that all the extracts were capable of scavenging the DPPH radical and the 2,2'-azinobis-(3-ethylbenzothiazoline-6-sulfonic acid) (ABTS) radical cation. The strongest antioxidant and reduction potential appeared in the extracts from seeds germinating under chill stress. As mentioned before, these extracts also contained the highest concentrations of phenolics. Positive correlation has been demonstrated between the reduction potential and antioxidant activity and the total content of phenolics. Yen and Chen [28], who examined different varieties of tea, found positive correlation between the total content of phenolic compounds in tea extracts and their reduction potential. In other studies conducted on soybean, wheat, sesame and sunflower seeds, positive relationships were discovered between the antioxidant properties of extracts and their content of phenoics [29].

## 3. Experimental Section 

### 3.1. Chemicals

All solvents used were of analytical grade unless otherwise specified. Methanol, acetone, hexanes, acetonitrile, potassium ferricyanide, FeCl_3_ × 6 H_2_O, and trichloroacetic acetic were acquired from the P.O.Ch. Company (Gliwice, Poland). Vanillin, Folin and Ciocalteu’s phenol reagent, 2,2'-diphenyl-1-picrylhydrazyl radical (DPPH•), 6-hydroxy-2,5,7,8-tetramethyl-chroman-2-carboxylic acid (Trolox), 2,2'-azinobis-(3-ethylbenzothiazoline-6-sulfonic acid) (ABTS), (+)-catechin, (−)-epicatechin, gallic acid, caffeic acid, *p*-coumaric acid, and ferulic acid were obtained from Sigma-Aldrich (Poznań, Poland). 

### 3.2. Plant Material

The experiments were conducted on *Vitis californica* supplied by Sandeman Seeds (Lalongue, France). 

### 3.3. Stratification and Germination under Normal and Chill Stress Conditions

Seeds were surface sterilized in 0.5% sodium hypochlorite for 20 min and washed with sterilized water. Stratification was conducted in a 1:1 mixture of sand and peat for four months at +4 °C. After stratification, 100 seeds were put into Petri-dishes of 140 × 25 mm on wood wool layers, soaked with 60 mL water and germinated. During germination in the dark, the plates were sealed with parafilm to prevent evaporation. Germination under optimal conditions (+25 °C) was conducted for 10 days. The seeds whose radicles were *ca.* 1 mm in length underwent further germination. The seeds were divided into two batches: one was germinated for two consecutive days under optimum conditions (+25 °C, sample NS); the other one germinated under chill stress (+10 °C, sample S), likewise for two days. Some of the seeds submitted to stress underwent recovery, by being germinated under optimum conditions (+25 °C, sample S + R) for the two following days. Once the set germination periods terminated, the germinating seeds were frozen in liquid nitrogen and stored at −80 °C until further analyses. 

### 3.4. Extract Preparation

Seeds were ground in a coffee mill and defatted with hexanes in a Soxhlet apparatus for 6–8 h. Phenolic compounds were then extracted from raw material using 80% (*v*/*v*) acetone at a solids to solvent ratio of 1:10 (*w*/*v*), at 50 °C for 30 min [30]. The extraction was carried out in Erlenmeyer flasks using a shaking water bath (Elpan 357, Wrocław, Poland). The extraction was repeated twice more, the supernatants were filtrated and combined, and the organic solvent was evaporated under vacuum at 40 °C in a Büchi rotary evaporator (Büchi Labortechnik AG, Flawil, Switzerland); the remaining aqueous solution was lyophilized. The prepared extract was stored at −20 °C until analysed.

### 3.5. Content of Total Phenolics

The content of total phenolics in the crude extracts was estimated using Folin and Ciocalteu’s phenol reagent [31]. (+)-Catechin was used as a standard in this work.

### 3.6. Condensed Tannins

The content of condensed tannins in the crude extract was determined using the modified vanillin assay [32] and BSA precipitation method [33]. Results were expressed as absorbance units at 500 nm per 1 g of extract (A_500_/g) and 510 nm per 1 g of extract (A_510_/g).

### 3.7. Trolox Equivalent Antioxidant Capacity

The Trolox Equivalent Antioxidant Capacity (TEAC) was determined by using the method of Re *et al*. [34]. For this method, ABTS•^+^ (2,2-azino-bis-3-ethylbenzothiazoline-6-sulfonic acid radical acation radical) solution was prepared by mixing an ABTS stock solution in water with 2.45 mM sodium persulfate. This mixture was allowed to stand with shaking for 12–16 h at room temperature in the dark until reaching a stable oxidative state. For analysis, the ABTS•^+^ stock solution was diluted with methanol to an absorbance of 0.720 at 734 nm. For the spectrophotometric assay, 2 mL of the ABTS•^+^ solution and 20 µL of the extract were mixed and the absorbance was determined at 734 nm at 37 °C for 10 min. The calibration curve was plotted by using Trolox standard. The results were expressed as mmol Trolox equivalent per g FW. 

### 3.8. Reducing Power

The reducing power of phenolics was determined as described by Oyaizu [35]. A suspension of extract in 1 mL of deionized water was mixed with 2.5 mL of 0.2 M phosphate buffer (pH 6.6) and 2.5 mL of 1% (*w*/*v*) potassium ferricyanide. The mixture was incubated at 50 °C for 20 min. Following this, 2.5 mL of 10% (*w*/*v*) trichloroacetic acid was added and the mixture was then centrifuged at 1750× *g* for 10 min. A 2.5-mL aliquot of the upper layer was mixed with 2.5 mL of deionized water and 0.5 mL of 0.1% (*w*/*v*) FeCl_3_. The absorbance of the mixture was measured at 700 nm with the spectrophotometer.

### 3.9. Scavenging of the 2,2'-Diphenyl-1-picrylhydrazyl (DPPH) Radical

The scavenging effect of phenolics from the extracts was monitored as described by Amarowicz *et al.* [31]. A 0.1 mL methanolic solution containing between 0.02 and 0.10 mg of extract was mixed with 2 mL of deionized water and then added to a methanolic solution of DPPH• (1 mM, 0.25 mL). The mixture was vortexed for 1 min, left to stand at room temperature for 20 min, and absorbance of the solution was then measured at 517 nm with the spectrophotometer.

### 3.10. High Performance Liquid Chromatography with Photodiode Array Detection (HPLC-PAD) Analysis of Catechins

Methanolic extract (10 mg) was dissolved in 1 mL of 80% (*v*/*v*) methanol and filtered through a 0.45 μm cellulose acetate filter (EMD Millipore, Billerica, MA, USA) before HPLC analysis. Catechins were analysed using a Shimadzu HPLC system (Shimadzu Corp., Kyoto, Japan) consisting of two LC-10AD pumps, SCTL 10A system controller and SPD-M 10A photodiode array detector. The chromatography was carried out using a pre-packed LiChrospher 100 RP-18 column (4 × 250 mm, 5 µm; Merck, Darmstadt, Germany). Elution for 50 min in a gradient system of 5%–40% acetonitrile in water adjusted to pH 2.5 with trifluoroacetic acid (TFA) was employed [18]; detector was set at 280 nm, injection volume was 20 µL and the flow rate was 1 mL/min. 

### 3.11. HPLC-PAD Analysis of Phenolic Acids

Phenolic acids (free and those liberated from soluble esters and from soluble glycosides) were isolated from the extracts according to the method previously described by Weidner *et al*. [36]. An aqueous suspension of the methanolic extract (200 mg in 20 mL) was adjusted to pH 2 with 6 M HCl, and free phenolic acids were extracted five times into 20 mL of diethyl ether using a separatory funnel. The ether extract was evaporated to dryness under vacuum at room temperature. The water solution was neutralised and then lyophilised. The residue was dissolved in 20 mL of 2 M NaOH and hydrolysed for 4 h under nitrogen atmosphere at room temperature. After acidification to pH 2 using 6 M HCl, phenolic acids released from soluble esters were extracted from the hydrolysate five times into 30 mL of diethyl ether. Nine milliliters of 6 M HCl were added to the water solution and the solution was placed in nitrogen atmosphere and hydrolyzed for 1 h in a boiling water bath. Phenolic acids released from soluble glycosides were separated from the hydrolysate five times into 45 mL of diethyl ether. After ether evaporation, the dry residue was dissolved in 2 mL of methanol and filtered through a 0.45 µm nylon filter. The sample was injected onto an HPLC column. The same Shimadzu HPLC system was employed. The conditions of the separations were as follows: prepacked LUNA C_18_ column (5 µm, 4.6 × 250 mm; Phenomenex, Torrance, Canada); mobile phase water-acetonitrile-acetic acid (88:10:2, *v/v/v*) [37]; flow rate of 1 mL/min; injection volume of 20 µL; the detector was set at 280 and 320 nm; oven temperature was 20 °C.

### 3.12. Statistical Analysis

All determinations were performed in this study in triplicate. Results are reported as mean and standard deviation (SD) values. Analyses of variance and Tukey’s studentised test were performed at level of *p* < 0.05 to evaluate the significance of differences among mean values.

## 4. Conclusions 

Under chill stress, the germinating seeds were characterized by the higher content of total phenolics, condensed tannins, catechins, gallic acid, and cafeic acid. The levels of *p*-coumoric and ferulic acids were found to have decreased. In the extracts obtained from a sample exposed to low temperature, increased antioxidant activity and reduction potential were also demonstrated. Tissue of the germinating seeds which underwent post-stress recovery were found to have less total phenolics. 
